# Thirty-Eight-Negative Kinase 1 Is a Mediator of Acute Kidney Injury in Experimental and Clinical Traumatic Hemorrhagic Shock

**DOI:** 10.3389/fimmu.2020.02081

**Published:** 2020-08-26

**Authors:** Rebecca Halbgebauer, Ebru Karasu, Christian K. Braun, Annette Palmer, Sonja Braumüller, Anke Schultze, Fabian Schäfer, Sarah Bückle, Alica Eigner, Ulrich Wachter, Peter Radermacher, Ranillo R. G. Resuello, Joel V. Tuplano, Kristina Nilsson Ekdahl, Bo Nilsson, Milena Armacki, Alexander Kleger, Thomas Seufferlein, Miriam Kalbitz, Florian Gebhard, John D. Lambris, Martijn van Griensven, Markus Huber-Lang

**Affiliations:** ^1^Institute of Clinical and Experimental Trauma Immunology, University Hospital Ulm, Ulm, Germany; ^2^Department of Pediatrics and Adolescent Medicine, University Hospital Ulm, Ulm, Germany; ^3^Institute for Anesthesiological Pathophysiology and Process Development, University of Ulm, Ulm, Germany; ^4^Simian Conservation Breeding and Research Center, Makati, Philippines; ^5^Department of Immunology, Genetics and Pathology, Uppsala University, Uppsala, Sweden; ^6^Centre of Biomaterials Chemistry, Linnaeus University, Kalmar, Sweden; ^7^Department of Internal Medicine I, University Hospital Ulm, Ulm, Germany; ^8^Department of Traumatology, Hand-, Plastic-, and Reconstructive Surgery, Center of Surgery, University Hospital Ulm, Ulm, Germany; ^9^Department of Pathology and Laboratory Medicine, University of Pennsylvania, Philadelphia, PA, United States; ^10^MERLN Institute for Technology-Inspired Regenerative Medicine, Department of Cell Biology-Inspired Tissue Engineering, Maastricht University, Maastricht, Netherlands

**Keywords:** trauma, injury, TNK1, AKI, complement, inflammation, IL-6, blood loss

## Abstract

Trauma represents a major socioeconomic burden worldwide. After a severe injury, hemorrhagic shock (HS) as a frequent concomitant aspect is a central driver of systemic inflammation and organ damage. The kidney is often strongly affected by traumatic-HS, and acute kidney injury (AKI) poses the patient at great risk for adverse outcome. Recently, thirty-eight-negative kinase 1 (TNK1) was proposed to play a detrimental role in organ damage after trauma/HS. Therefore, we aimed to assess the role of TNK1 in HS-induced kidney injury in a murine and a *post hoc* analysis of a non-human primate model of HS comparable to the clinical situation. Mice and non-human primates underwent resuscitated HS at 30 mmHg for 60 min. 5 h after the induction of shock, animals were assessed for systemic inflammation and TNK1 expression in the kidney. *In vitro*, murine distal convoluted tubule cells were stimulated with inflammatory mediators to gain mechanistic insights into the role of TNK1 in kidney dysfunction. In a translational approach, we investigated blood drawn from either healthy volunteers or severely injured patients at different time points after trauma (from arrival at the emergency room and at fixed time intervals until 10 days post injury; identifier: NCT02682550, https://clinicaltrials.gov/ct2/show/NCT02682550). A pronounced inflammatory response, as seen by increased IL-6 plasma levels as well as early signs of AKI, were observed in mice, non-human primates, and humans after trauma/HS. TNK1 was found in the plasma early after trauma-HS in trauma patients. Renal TNK1 expression was significantly increased in mice and non-human primates after HS, and these effects with concomitant induction of apoptosis were blocked by therapeutic inhibition of complement C3 activation in non-human primates. Mechanistically, *in vitro* data suggested that IL-6 rather than C3 cleavage products induced upregulation of TNK1 and impaired barrier function in renal epithelial cells. In conclusion, these data indicate that C3 inhibition *in vivo* may inhibit an excessive inflammatory response and mediator release, thereby indirectly neutralizing TNK1 as a potent driver of organ damage. In future studies, we will address the therapeutic potential of direct TNK1 inhibition in the context of severe tissue trauma with different degrees of additional HS.

## Introduction

Trauma has been, is (1), and will be a major individual burden and socioeconomic load worldwide. After severe trauma, hemorrhagic shock (HS) represents an important driving mechanism of systemic inflammation, barrier breakdown, organ dysfunction, and adverse outcome (2, 3). A frequently failing organ post-trauma is the kidney. Acute kidney injury (AKI), defined as an abrupt decrease in kidney function, develops in around 25% of all severely injured patients and in more than 40% as a result of HS (4) with a high risk of infectious complications and increased lethality (5, 6). However, the underlying mechanisms of traumatic HS-induced AKI are still not well-understood.

Besides mounting a posttraumatic systemic inflammatory response with enhanced cytokine plasma levels such as interleukin 6 (IL-6), activation of the coagulation and complement systems may be involved in the development of posttraumatic AKI (2, 7). The central complement component C3, which is mainly produced in the liver and kidneys (8), is strongly activated after trauma resulting in enhanced systemic levels of the potent anaphylatoxin C3a which correlates with the injury severity score (ISS), development of septic complications, and lethal outcome (9, 10). In the kidneys, C3a can alter the function of human mesangial cells toward a more profibrotic phenotype (11). During the systemic inflammatory response in *Escherichia coli*-induced sepsis of non-human primates, blockade of C3 and its activation products by compstatin resulted in structural and functional renoprotection (12). In the setting of (traumatic-) HS in non-human primates, we could recently demonstrate that blockade of C3 by the compstatin analog Cp40 beneficially modulated the inflammatory response on a systemic and organ-specific level, and prevented organ injury while improving urine output (13). However, it remains in the dark what mechanism is involved in renoprotection beyond blockade of C3.

Concerning mechanisms activating C3 during traumatic-HS, Szebeni et al. proposed in a pig model of HS that lactic acid, lipopolysaccharide (LPS), and natural antibodies bound to hypoxic cellular surfaces may activate C3 resulting in complement consumption and anaphylatoxin generation which may aggravate shock pathophysiology (14). Focusing on the kidneys, blockade of the classical complement activation pathway further upstream by a C1 inhibitor treatment resulted in lower blood levels of urea nitrogen and creatinine after HS in pigs (15). Furthermore, in a rodent HS model, C3 blockade rescued vascular hyporeactivity (16). In experimental multi-organ dysfunction following a double hit of low-dose LPS and HS, complement activation and deposition of C3 cleavage products were detected in various organs including the kidneys (17).

Organ protection during traumatic HS can in principle be caused by various mechanisms including therapeutic strategies addressing damage-associated molecular patterns (18), immunomodulation (19), coagulation (20), oxidative stress (21), hibernation programs (22), endothelial dysfunction and barrier sealing (2), and others.

Recently, we proposed a novel mechanism of organ (gut) protection in this context by deletion of thirty-eight-negative kinase 1 (TNK1), also termed tyrosine kinase non-receptor 1 (23). The functional features of TNK1 are still under investigation, but it seems that TNK1 expression results in STAT3 phosphorylation, p65 nuclear translocation, and generation of IL-6 as well as TNF. Consequently, TNK1 is involved in immunomodulation and apoptotic processes (24) during inflammation and also after trauma (23). Based on these close links between TNK1 and acute inflammatory processes as well as improved renal function after early complement inhibition, TNK1 represents a promising candidate to be involved in the pathophysiology of HS-induced AKI. However, TNK1 seems to be regulated mainly by expression (23). It is only moderately expressed in kidneys (25) which theoretically could be changed by a traumatic and/or hypoxic microenvironment such as during HS, in which condition enhanced levels of the inflammatory mediators C3a or IL-6 have been found.

Based on recent findings of intestinal mechanisms in development of multiple-organ failure, TNK1 has been proposed as a “gateway to multiple-organ failure” (23, 26). Although no direct interactions have been found so far between TNK1 and central complement factors, it was tempting to speculate that C3 activation contributes to AKI in concert with TNK1 early after trauma.

We therefore hypothesized that (i) TNK1 expression in renal tissue is increased by severe traumatic HS and (ii) that this detrimental upregulation can be prevented by blocking activation of the central complement component C3 with downstream systemic generation of pro-inflammatory mediators. To investigate a possible TNK1 impact on kidneys in the setting of trauma-HS-induced AKI, we tested the effects translationally from a well-established murine (27) and non-human primate traumatic-HS model (13) toward clinically meaningful monitoring of samples from severely injured patients with HS.

## Materials and Methods

### HS in Mice

Twelve male C57BL/6J mice aged 8 to 12 weeks (Charles River Wiga GmbH, Sulzfeld, Germany) with a mean body weight of 29.5 *g* (±0.4 *g*) had access to food and water *ad libitum*. Animals were randomly divided into HS animals (*n* = 8) and untreated control animals (Ctrl, *n* = 4). Mice were anesthetized with 2.5% sevoflurane (Sevorane, Abbott, Wiesbaden, Germany)/97.5% oxygen, which was continued during the hemorrhagic procedure and during the whole observation period. 0.05 mg/kg body weight buprenorphine was administered subcutaneously for analgesia. The study protocol was approved by the University Animal Care Committee and the Federal Authorities for Animal Research, Tübingen, Germany (approval no. 1194), and all experiments were performed in adherence to the National Institute of Health Guidelines for the use of laboratory animals.

Shock was induced as described previously (27, 28). Briefly, a micro-catheter (Föhr Medical Instruments, Seeheim/Ober-Beerbach, Germany) was inserted into the femoral artery for monitoring the blood pressure and for induction of a controlled blood loss to simulate HS. A jugular vein catheter was applied to fluid resuscitate animals and apply catecholamine infusion if indicated. Mice were bled for 5 to 10 min until they reached a mean arterial pressure (MAP) of 30 mmHg (±5 mmHg) which was kept stable for 60 min by rebleeding if indicated by the continuous monitoring. Animals were resuscitated with a balanced electrolyte solution (Jonosteril^®^, Servoprax, Wesel, Germany) via the jugular vein at the 4-fold volume of the drawn blood over 60 min with a target blood pressure of 50 mmHg. After this procedure, animals were observed and monitored for further 3 h. Anesthesia and norepinephrine (0.01–0.12 μg/kg/min; Sanofi, Frankfurt am Main, Germany) support were adjusted according to a preset protocol to maintain a MAP of 50 mmHg. 3 h after resuscitation, EDTA-anticoagulated blood was drawn by cardiac puncture and centrifuged at 800 × *g* and 4°C for 5 min; supernatants were centrifuged again at 13,000 × *g* and 4°C for 2 min. Urine was collected, and urine and plasma samples were stored at −80°C until analysis. IL-6 (IL-6, BD Biosciences, Heidelberg, Germany), TNK1 (EIAab, Wuhan, China), neutrophil gelatinase-associated lipocalin (NGAL, LifeSpan BioSciences, Seattle, United States), as well as kidney injury molecule-1 (KIM-1, R&D Systems, Minneapolis, United States) were determined via enzyme-linked immunosorbent assay (ELISA). In order to correct for fluids administered, ELISA results were normalized to total plasma protein. Following final blood withdrawal, kidneys were harvested for histological analysis (see below) or snap-frozen in liquid nitrogen to be analyzed using qPCR. Plasma creatinine was measured as described previously (27).

### HS in Non-human Primates

In this study, we performed a *post hoc* analysis of samples taken from a previous study (13). Eight male cynomolgus monkeys (*Macaca fascicularis*) weighing 4.8 ± 0.4 kg, aged 4–5 years were divided into two groups (Cp40 and vehicle control, *n* = 4). The study protocol was approved by the IACUC of the Simian Conservation Breeding and Research Center in Makati City, Philippines (Study *SIC*-120 approval number 2013-02) and has been described in detail elsewhere (13). All experiments were performed in accordance with the National Institutes of Health Guide for the Care and Use of Laboratory Animals.

Animals were anesthetized, intubated and ventilated, catheterized in femoral arteries and veins, and a urine catheter was placed via the urethra. After 15 min stabilization time, HS was induced by blood withdrawal from one femoral artery until a MAP of 30 mmHg was reached or until 45% of total blood volume was withdrawn. Blood pressure was kept at 30 mmHg for 1 h; then, animals were resuscitated with Ringer’s solution at four times the withdrawn blood volume within 30 min. During the following observation period, intravenous infusion of Ringer’s was maintained at 10 mL/kg/h. Norepinephrine was given when the MAP fell below 60 mmHg; glucose levels were controlled. Animals were treated with either the compstatin analogon Cp40 (3 mg/kg in 0.9% NaCl) or vehicle (0.9% NaCl) 30 min after shock induction by bolus injection; afterward, a continuous infusion of 4 μg/kg/min Cp40 in 0.9% NaCl was administered until the end of the experiment spanning over 5 h after induction of HS. In the vehicle group, the same amount of 0.9% NaCl was infused. At the end of observation, kidneys were collected for histological analysis. Three healthy animals served as controls.

### Immunohistochemical Staining of TNK1 and TUNEL Assay

Non-human primate and murine kidney samples were fixed in 3.7% formaldehyde in PBS (Fishar), dehydrated, and embedded in paraffin. 4 μm sections were cut, rehydrated, and stained using a monoclonal anti-TNK1 antibody (LSBio) at a dilution of 1:100. For detection, the Dako real detection Alkaline phosphatase Kit (Dako) was employed. Slides were visualized using a Zeiss Axio Imager A1 microscope with five randomly chosen field of views per animal and region in 200-fold magnification and intensity of expression was quantified using ZEN pro software (Zeiss). For the non-human primate samples, terminal deoxynucleotidyl transferase dUTP nick end labeling (TUNEL) staining was performed in order to quantify DNA strand breaks. After sectioning and rehydration, tissues were digested in proteinase K (Roche, Basel, Switzerland) for 15 min at 37°C. Subsequently, samples were stained using the *in situ* Cell Death Detection Kit, TMR red (Roche) according to the manufacturer’s instructions and counterstained with DAPI. After staining, positive events per field of view in 100-fold magnification were counted in five randomly selected regions per animal using the Zeiss Axio Imager A1 microscope.

### Quantitative Real-Time PCR

RNA was isolated from snap-frozen mouse kidneys using the RNeasy Mini Kit (Qiagen, Hilden, Germany) and cDNA was synthesized employing the AffinityScript QPCR cDNA Synthesis Kit (Agilent, Waldbronn, Germany). QPCR was performed using primers for Tnk1 and Gusb2 as a housekeeping gene (Qiagen) and the Brilliant II SYBR Green qPCR Master Mix (Agilent). Differences in expression were calculated employing the ΔCT method.

### Clinical Study in Severely Injured Patients

A prospective clinical study was conducted in thirteen patients after severe polytrauma (ISS ≥ 22) with different degrees of blood loss that were admitted to the emergency room of University Hospital Ulm between May 2015 and July 2016. The study protocol was approved by the Independent Local Ethics Committee of the University of Ulm (approval number 94/14). The study was registered on ClinicalTrials.gov, identifier NCT02682550, and was performed in accordance with the Declaration of Helsinki and its recent modifications. Exclusion criteria were age < 18 years, pregnancy, infection with the human immunodeficiency virus, cardiogenic shock as the primary underlying disease, underlying hematologic disease, cytotoxic therapy given within the previous 6 months, and the presence of rapidly progressing underlying disease anticipating death within the next 24 h. Eight healthy volunteers served as a control group. Before inclusion, written informed consent was obtained from all patients and volunteers; if the patient was incapable of making decisions because of intubation, sedation or altered mental status, informed consent was obtained directly after recovery or from the legal representative after appointment. EDTA blood was drawn at the time of admission (0 h) and 8 h, 24 h, 48 h, 5 days, and 10 days after trauma with a maximum divergence from time points of ±10%, transported on ice, and centrifuged immediately at 2200 × *g* and 4°C for 10 min. Supernatants were aliquoted on ice and stored at −80°C until further analysis. ELISA analysis was performed for TNK1 (Aviva Systems Biology, San Diego, United States), NGAL, IL-8 (both R&D Systems, Minneapolis, United States), IL-6 (BD Biosciences, Heidelberg, Germany), and C3a (Quidel, San Diego, United States) and results were normalized to total plasma protein. Clinical data for statistical correlation such as the initial number of transfused erythrocyte concentrates and serum creatinine levels were collected retrospectively from patients’ records.

### Murine Distal Convoluted Tubule Cell Line

209/MDCT cells (CRL-3250™, ATCC^®^, Manassas, United States) were grown at 37°C and 5% CO_2_ in a 1:1 mixture of Dulbecco’s Modified Eagle Medium and Ham’s F-12 Nutrient Mixture (Thermo Fisher Scientific, Waltham, United States) with 5% fetal bovine serum (PAN™ Biotech, Aidenbach, Germany) and 1% penicillin-streptomycin (Thermo Fisher Scientific, Waltham, United States) and subcultivated at approx. 80% confluence 2–3 times a week. Cells were seeded on the upper side of a Transwell^®^ Polyester Membrane Insert (pore size 0.4 μm, area 0.33 cm^2^, Corning, New York, United States) coated with collagen (Stem Cell™ Technologies, Vancouver, Canada) at 1 × 10^5^ cells/cm^2^ and medium was changed daily. On day three after seeding, cells were washed and stimulated with human C3a (1 μg/ml, Complement Technology, Tyler, United States), IL-6 (0.5 ng/ml, Biomol, Hamburg, Germany), or LPS (1 μg/ml, Sigma-Aldrich, Steinheim, Germany) in phenol red-free medium (Thermo Fisher Scientific, Waltham, United States) for 24 h. Dosages for C3a and IL-6 were chosen according to concentrations measured in human patients after severe injury as previously described (29). The concentration for LPS used was chosen according to data from another previous study (28). Unstimulated cells served as control. Afterward, basal medium was replaced with fresh phenol red-free medium; 4 kDa FITC-dextran (10 μg/ml, Sigma-Aldrich) in medium without phenol red was added apically and incubated at 37°C for 1 h. After incubation, the fluorescence (485 nm/530 nm) in basolateral media was measured in duplicate using a Fluoroskan Ascent^®^ (Thermo Fisher Scientific). For calculation of absolute FITC-dextran concentrations, a standard series was prepared from the FITC-dextran stock. For Western Blot analyses, cells were seeded on collagen-coated 12-well plates at 1 × 10^5^ cells/cm^2^ and medium was changed daily. On day 2 after seeding, cells were stimulated as described above for 6 h. Cells were lysed, centrifuged, and supernatants were stored at −80°C. For TNK1 detection, 30 μg of protein from each sample were denatured and sodium dodecyl sulfate polyacrylamide gel electrophoresis was performed in Mini-PROTEAN TGX Stain-Free Gels (Bio-Rad, Hercules, United States). After blotting, TNK1 expression was detected using an anti-mouse TNK1 Rabbit Polyclonal Antibody (1:2000; Proteintech, Rosemont, United States) and an anti-rabbit IgG HRP-linked secondary antibody (1:1500; Cell Signaling, Danvers, United States). TNK1 expression was visualized employing a ChemiDoc™ XRS Molecular Imager (Bio-Rad, Hercules, United States) and normalized to total membrane protein.

### Statistical Analysis

For statistical analysis, SigmaPlot version 14 (Systat Software, Inc.) was used. All values are expressed as means ± SEM. Data in two groups were compared using Welch’s *t*-test as equality of variances could not be assumed. Data in more than two groups were compared by one-way ANOVA followed by multiple Student-Newman–Keuls *post hoc* analysis in case of normal distribution and by Kruskal–Wallis one-way ANOVA on ranks with Dunn’s *post hoc* testing for non-normally distributed data. Correlation analyses were performed using Pearson product moment test. Results were considered significant when *p* ≤ 0.05.

## Results

### Systemic Inflammatory Reaction in HS Shock Across Various Species

**FIGURE 1:**
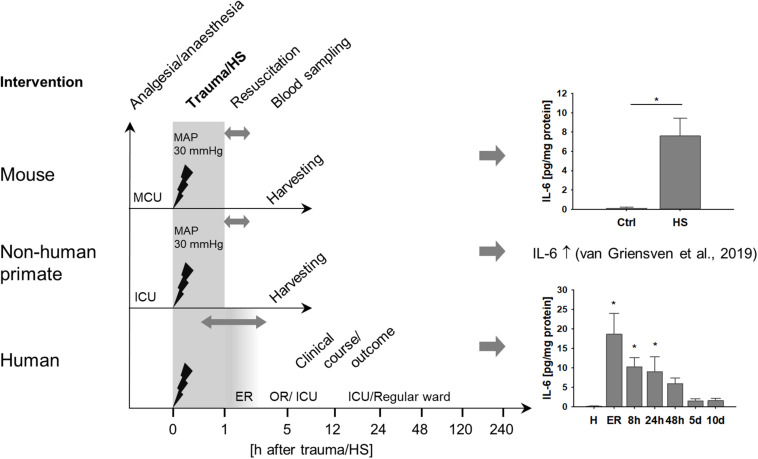
Systemic inflammation induced by hemorrhagic shock (HS) across various species. Mice and non-human primates were anesthetized and underwent severe hemorrhage at a mean arterial pressure (MAP) of 30 mmHg for 60 min. After resuscitation with crystalloids, animals were monitored until organ harvesting 5 h after shock induction. Mice demonstrated significantly increased plasma interleukin 6 (IL-6) after HS compared to healthy controls (ctrl). *p < 0.05, *n* = 4 ctrl, and *n* = 8 HS. As has been described elsewhere, HS in non-human primates induced a pronounced systemic pro-inflammatory response reflected by significantly increased plasma IL-6 (13). In a clinical prospective observational study, severely injured patients were monitored beginning upon arrival at the emergency room (ER) at fixed time intervals until day 10 after injury. Similar to mice and non-human primates, patients demonstrated distinct increases in plasma IL-6, especially during the first 24 h after trauma. *p < 0.05 vs healthy (H), *n* = 8 healthy, *n* = 11–13 polytrauma patients; ER, emergency room; ICU, intensive care unit; MCU, mouse intensive care unit; OR, operating room.

We analyzed the role of TNK1 in hemorrhage-induced kidney injury across various species. Figure 1 depicts the study design of murine and ape HS, closely modeling traumatic-HS in a prospective observational study in polytraumatized patients. Strong activation of a systemic inflammatory response was present in all three species in response to severe injury and/or hemorrhage as represented by significantly increased IL-6 concentrations in mice, non-human primates, and humans (Figure 1).

### Kidney Injury in Murine HS

**FIGURE 2:**
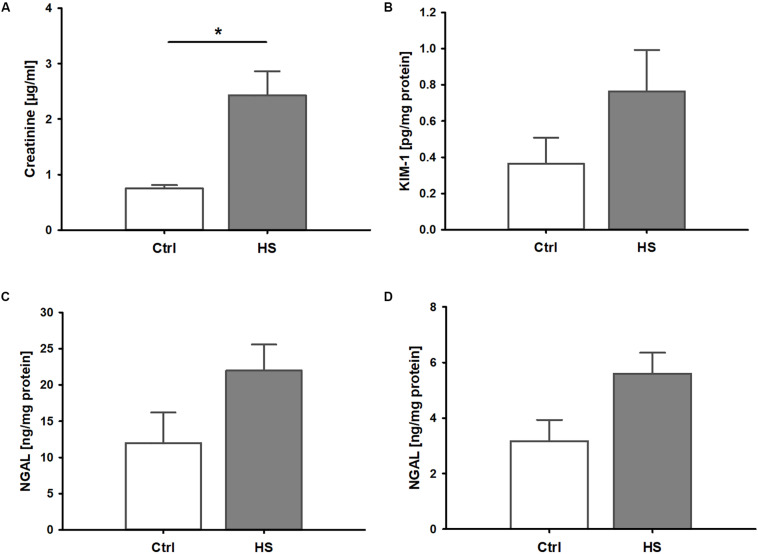
Kidney injury and dysfunction in murine HS. **(A)** Mice after hemorrhage (30 mmHg, 60 min) and resuscitation (HS) demonstrated significantly increased plasma creatinine compared to healthy controls (Ctrl). Plasma kidney injury molecule 1(KIM-1, **B**) and neutrophil gelatinase-associated lipocalin (NGAL) in plasma **(C)** and urine **(D)** were elevated by trend, but not significantly, in animals after HS. *p < 0.05; *n* = 4 ctrl, and *n* = 8 HS for **(A–C)**; *n* = 3 ctrl, *n* = 8 HS for **(D)**.

Mice displayed signs of kidney injury and functional impairment 3 h after reperfusion. Blood creatinine levels were significantly increased in HS animals compared to controls (Figure 2A). Furthermore, we observed a trend to elevated plasma levels (although not significant) of kidney injury markers such as KIM-1 (Figure 2B) and NGAL (Figure 2C) as well as slightly higher urinary NGAL concentrations (Figure 2D) in animals after resuscitated HS.

**FIGURE 3:**
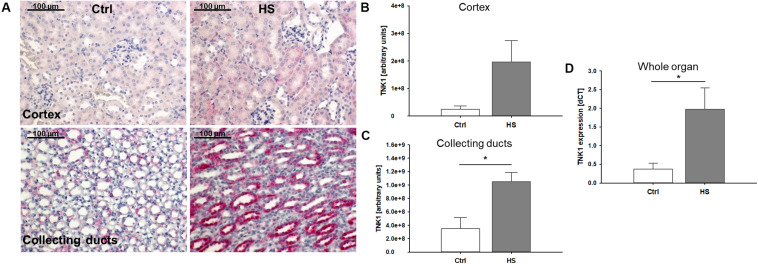
Non-receptor tyrosine-protein kinase 1 (TNK1) expression in murine kidneys after HS. TNK1 expression in kidneys of control (Ctrl) mice and animals after hemorrhagic shock (HS) was assessed by immunohistochemical staining and semiquantitative analysis. While there was only a modest increase in TNK1 expression (stained in red; nuclei were counterstained with hematoxylin) in the renal cortex **(A,B)**, expression was significantly higher in collecting ducts of animals after HS **(A,C)** compared to controls. Quantitative real-time PCR revealed a significant increase in TNK1 mRNA in whole tissue lysates **(D)**. *p < 0.05; *n* = 4 Ctrl, and *n* = 4 HS for **(B,C)**; *n* = 4 Ctrl, *n* = 7 HS for **(D)**.

In histological analyses of different kidney regions, we found a slight increase in TNK1 expression in the renal cortex, especially in tubules while expression in glomerular cells remained low (Figures 3A,B). Furthermore, significantly increased TNK1 levels were detected in collecting ducts (Figures 3A,C) in mice after HS. In lysates of whole kidney samples, we found an overall significantly higher gene expression of TNK1 in animals after hemorrhage compared to controls (Figure 3D).

### Role of Systemic Inflammation and TNK1 in HS-Induced Kidney Injury

**FIGURE 4:**
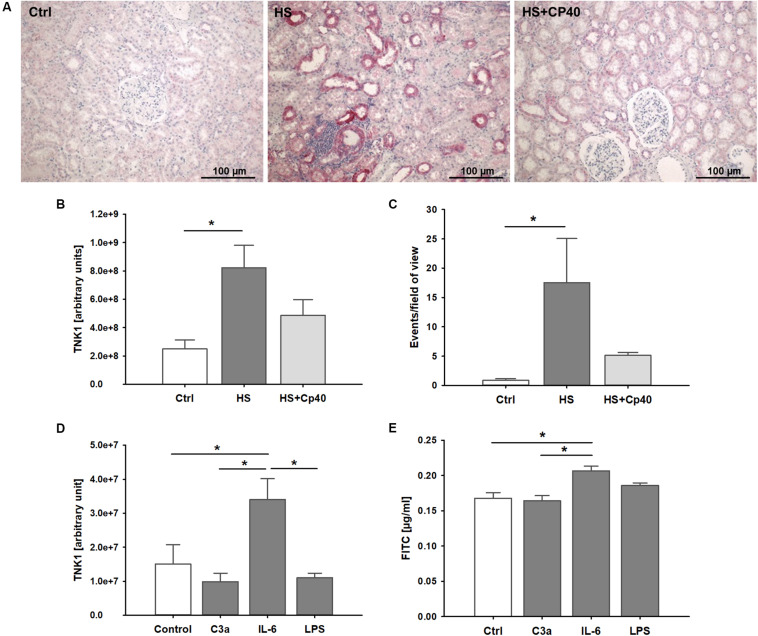
Role of non-receptor tyrosine-protein kinase 1 (TNK1) in hemorrhage-induced kidney injury. TNK1 expression and cellular apoptosis were analyzed in kidneys from non-human primates **(A–C)**. **(A)** TNK1 was stained by immunohistochemistry (TNK1 staining in red; nuclei were counterstained with hematoxylin) in non-human primate kidneys after hemorrhagic shock with placebo treatment (HS) or HS and treatment with the complement inhibitor Cp40 (HS + Cp40) as well as in healthy control animals (Ctrl). Quantification of stained tissues revealed a significant increase in TNK1 expression in HS animals compared to controls which was abolished by Cp40 treatment **(B)**. A TUNEL assay showed similar results with a significant increase in apoptotic events in kidneys after hemorrhage which was prevented by complement blockade using Cp40 **(C)**; *p < 0.05, *n* = 3 Ctrl, *n* = 4 HS, *n* = 4 HS + Cp40 for **(B,C)**. In murine distal convoluted tubule cells **(D,E)**, TNK1 expression was significantly increased after stimulation with interleukin 6 (IL-6), but not altered after incubation with activated complement component 3 (C3a), or lipopolysaccharide (LPS, **D**). Cellular permeability for 4 kDa FITC-labeled dextran in a transwell chamber was significantly increased after stimulation with IL-6, but not with C3a or LPS **(E)**. The data in **(D)** and **(E)** are representative of two independent experiments. *p < 0.05, *n* = 5 for **(D,E)**.

In kidneys from non-human primates after severe hemorrhage, we observed significantly higher TNK1 expression especially in distal tubule epithelial cells and collecting duct cells which was abolished after systemic complement C3 blockade (Figures 4A,B). TUNEL staining revealed significantly increased events of DNA strand breaks in animals after HS, but not after therapeutic application of Cp40 (Figure 4C). In *in vitro* assays in a murine distal tubule cell line, we aimed to determine the responsible pro-inflammatory molecules. Stimulation with activated complement component C3a, IL-6, and LPS revealed that C3a and LPS had no effect while IL-6 significantly increased TNK1 expression (Figure 4D). Furthermore, permeability of murine distal tubule cells in a Transwell chamber for a 4 kDa molecule was significantly increased after incubation with IL-6, but not with C3a or LPS (Figure 4E).

### Inflammation, TNK1, and Kidney Injury in Severely Injured Patients

**FIGURE 5:**
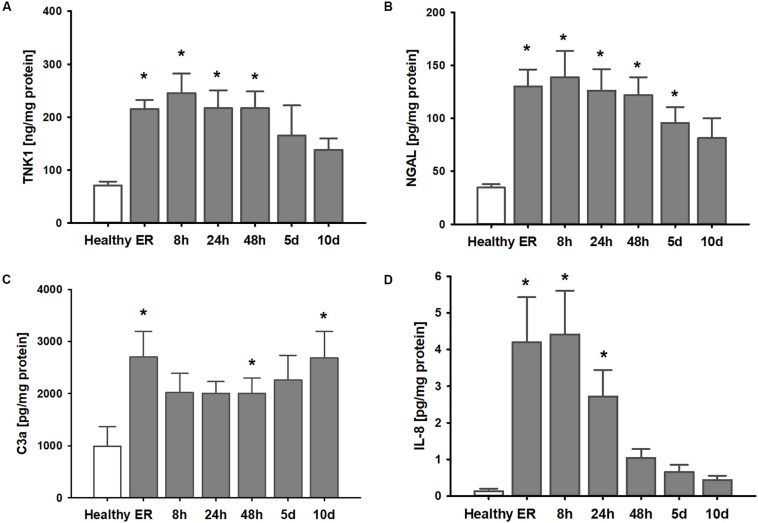
Elevation of systemic TNK1 and plasma markers of organ damage and inflammation in severely injured patients. In polytraumatized (PT) patients, plasma concentrations of **(A)** non-receptor tyrosine-protein kinase 1 (TNK1) and **(B)** of the kidney injury marker neutrophil gelatinase-associated lipocalin (NGAL) were significantly increased starting upon arrival at the emergency room (ER) and remained elevated throughout the first days after injury compared to healthy volunteers (healthy). Plasma concentrations of the activated central complement component C3 (C3a) were increased in trauma patients throughout the entire observation period **(C)**. Systemic interleukin 8 (IL-8) was significantly increased especially during the first 24 h after trauma **(D)**. *p < 0.05 vs. healthy, *n* = 7-8 healthy, and *n* = 11-13 PT patients.

Clinical parameters of the thirteen patients after polytrauma and eight healthy volunteers are shown in Table 1. Healthy volunteers did not demonstrate any signs of kidney disease at the time of sample collection (serum creatinine 1.01 ± 0.04 mg/dl). In patients, plasma TNK1 was increased significantly starting at the first blood withdrawal in the emergency room and lasting until 48 h after injury; concentrations did not reach the levels found in healthy volunteers until 10 days after injury (Figure 5A). The same pattern was documented for the plasma kidney damage marker NGAL which was significantly increased starting upon arrival at the emergency room until day 5 post trauma (Figure 5B). The complement activation product and anaphylatoxin C3a was significantly increased in plasma from patients admitted to the hospital and remained elevated until the end of the observation period (Figure 5C). IL-8 showed a similar pattern to IL-6 values (Figure 1) with the highest concentrations measured immediately after injury (Figure 5D). There was no significant correlation between age and TNK1 levels for patients or healthy volunteers (data not shown).

**TABLE 1:** Data are shown as mean ± SEM.

	Polytrauma	Healthy volunteers	*p*-value
Sex (m/f)	10/3	5/3	n.s.
Age (years)	48.35	33.9 ± 3.9	n.s.
ISS (points)	34.92	n/a	
Initial creatinine (mg/dl)	1.20.1	1.0 ± 0.05	n.s.
Initial hematocrit	0.30.02	n/a	
RBCs < 24 h (units)	8.22.6	n/a	
TASH score (points)	12.61.7	n/a	

*Ages and creatinine levels were compared using Student’s *t* test; sexes were compared using Fisher’s exact test. ISS, Injury Severity Score; RBC, red blood cell concentrates; TASH, Trauma-Associated Severe Hemorrhage.*

**FIGURE 6:**
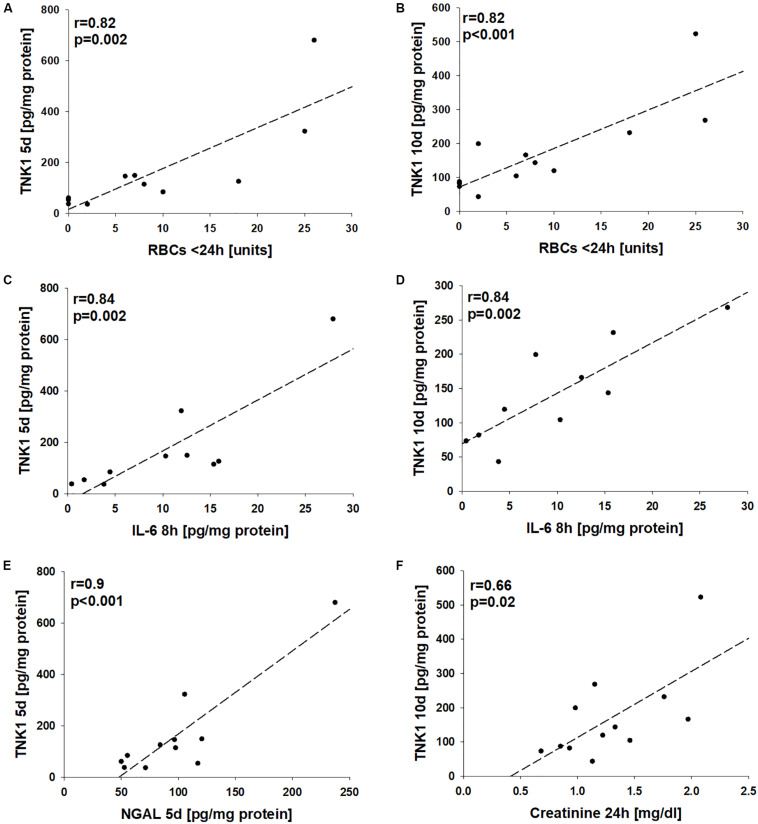
Association of non-receptor tyrosine-protein kinase 1 (TNK1)-mediated kidney injury with systemic inflammatory markers. Correlation analyses were performed for plasma values of TNK1 with parameters related to hemorrhagic shock (HS), inflammation, and kidney dysfunction in severely injured patients. The numbers of applied red blood cell concentrates (RBC) during the first 24 h were significantly correlated with plasma TNK1 5 days **(A)** and 10 days after trauma **(B)**. Similarly, early levels of interleukin 6 (IL-6, 8 h after injury) significantly correlated with TNK1 5 days **(C)** and 10 days after trauma **(D)**. The plasma kidney injury marker neutrophil gelatinase-associated lipocalin (NGAL) was significantly correlated with TNK1 levels on day 5 **(E)**. Furthermore, plasma creatinine 24 h after injury was associated with TNK1 on day 10 **(F)**. *r*, Pearson correlation coefficient.

Correlation analyses revealed significant correlations between the number of administered units of red blood cells during the first 24 h after trauma with late plasma TNK1 levels (both *r* = 0.82, Figures 6A,B). Furthermore, there were significant correlations between early plasma IL-6 levels with plasma TNK1 on days 5 and 10 (both *r* = 0.84, Figures 6C,D). Late NGAL plasma concentrations were significantly correlated with plasma TNK1 on day 5 (*r* = 0.9, Figure 6E). Plasma creatinine 24 h after injury was significantly related to TNK1 on day 10 (Figure 6F).

## Discussion

HS has been shown to function as a major contributor to the development of AKI after severe tissue trauma (3). In the present study, we have employed a translational approach based on an established murine HS model (27), a subanalysis of a recently performed non-human primate study on HS (13), and a polytrauma cohort with HS in clinical reality. Both preclinical HS models used a similar pressure-controlled (30 mmHg ± 5 mmHg) shock period of 60 min and a resuscitation protocol applying four times the withdrawn blood volume by a balanced electrolyte solution and norepinephrine support if needed over a comparable post-shock observation time. In both cases, early signs of AKI developed within the first 4 h after shock with enhanced levels of creatinine and kidney damage markers. In the clinical cohort, blood transfusions were conducted usually only after 60 min post trauma/HS, normally because no blood products are given during the preclinical phase according to most German emergency protocols (30). However, the patients received balanced electrolyte solutions and norepinephrine support if needed. Thus, the initial treatment algorithms in our animal models, although with some minute differences, may to a large extent be of translational validity. As a limitation of our study, animals were not ventilated during the procedure, avoiding presumable ventilation effects on renal blood flow and glomerular filtration rate. The systemic inflammatory response after trauma-HS was evident in all three cases as reflected by enhanced plasma levels of IL-6 (Figure 1); mean IL-6 levels in non-human primates during the time course after HS ranged between 10–150 pg/ml and were significantly reduced in animals after therapeutic complement blockade by Cp40 as has been published elsewhere (13). In the clinical setting, IL-6 has been established as early overall damage and inflammatory response marker with some prognostic value for the outcome after severe injury (31). Whether IL-6 functions only as a monitoring marker or is also causatively involved in driving the pathophysiology of AKI development is the objective of current research. Both IL-6 and NGAL determined shortly after surgical tissue injury inflicted by major cardiac surgery were independent predictors of AKI in the postoperative course (32). In murine kidney ischemia and reperfusion injury, anti-IL-6 therapy by gene delivery was capable of improving renal function and of decreasing NGAL concentrations, indicating some causal involvement of IL-6 (33). IL-6 also seems to have anti-inflammatory effects via upregulation of splenic IL-10 and thereby inhibiting AKI-induced remote organ injury such as lung inflammation (34). Here, we report that IL-6 was able to induce TNK1 expression in a kidney cell line, which so far has been barely found to be regulated. These *in vitro* data are supported by *in vivo* findings of enhanced IL-6 plasma levels correlated with increased TNK1. The role of TNK1 in kidneys has not been evaluated so far. Recently, we described the detrimental effects of enhanced TNK1 expression in the gut as a driver of multiple-organ failure (23, 26). In accordance, we found here for the first time an enhanced expression of TNK1 in the kidneys after traumatic-HS. The involved pathways, triggered by TNK1, are likely STAT3 phosphorylation, nuclear translocation of p65, and/or release of IL-6 and TNF as recently reported for the intestine (23), which as a limitation of the study have not been further defined in detail. However, based on present data it is tempting to speculate that enhanced IL-6 plasma levels may be at least in part a result of upregulated TNK1. Vice versa, we show here that IL-6 can upregulate renal TNK1 expression. Of note, traumatic HS-induced upregulated renal TNK1 was inhibited by blockade of the central complement component C3. As recently reported, C3 blockade by the small peptide inhibitor Cp40 was able to prevent signs of lung, intestinal, and kidney injury and to inhibit the systemic inflammatory response after (traumatic) HS (13). Here, it seems that Cp40 was capable of inhibiting upregulation of TNK1 expression and induction of apoptosis in the kidneys. However, we did not observe a direct effect of activated C3 on TNK1 expression in murine distal convoluted tubule cells; as a minor limitation, murine cells were stimulated with human C3a and IL-6 which may require different effective concentrations than human cell lines. Still, it is more likely that the *in vivo* demonstrated renoprotective effects of Cp40 in regard to TNK1 are mechanistically caused rather indirectly via Cp40-induced reduction of the systemic inflammatory reaction and prevention of extensive IL-6 release. In support, enhanced plasma levels of IL-6 after HS were indeed almost normalized after treatment with Cp40 (13), indicating the abolishment of IL-6 as a potent inducer of systemic and renal TNK1 expression by effective C3 blockade. In this regard, the complex role of IL-6 classical and *trans*-signaling may be of high interest in coming studies of HS-induced AKI. As a limitation, besides including data from both male and female patients, we have performed our animal studies in relatively small groups of animals and exclusively in male animals due to local restrictions in animal numbers (3R principles) and/or sexes. It is therefore vital for future pre-clinical studies to assess the influence of sex as well as age and comorbidities when evaluating the benefits of complement inhibition in traumatic HS.

So far, TNK1 has not been described in the context of human tissue injury and HS. Here, we have detected enhanced plasma levels of TNK1 in severely injured polytrauma patients (ISS ≥ 22) with HS as early as in the emergency room lasting for up to 48 h post-injury. Furthermore, large-volume blood transfusion during the first 24 h after injury, early blood IL-6 levels, as well as systemic markers of kidney injury and dysfunction were correlated with higher blood TNK1 concentrations after 5 and 10 days. As a limitation, we did not perform non-linear regression analyses which might in part provide a more suitable approach to represent the biological relationship between clinical parameters and TNK1 expression. Although from these human data no causative conclusion can be drawn, TNK1 may represent an interesting candidate for immunomonitoring because its upregulation seems to contribute to MODS (23) via its catalytic activity since the expression is the major way of regulating the catalytic activity of this kinase. Thus, TNK1 is a possible future target to modulate the post-HS response either directly or indirectly. For example, TNK1 affects the integrity of both adherens and tight junctions (such as E-cadherin and claudins) in the intestine. It could be speculated that TNK1 may also impair the blood-urine barrier in the kidneys. In this context, IL-6 enhanced the permeability of kidney epithelium *in vitro*, which again might be influenced by therapeutic blockade of C3 activation.

A further possible mechanism how TNK1 could alter kidney function regards the repair of damaged tubuli. In non-human primates, we could confirm results from previous studies showing that AKI induces tubular apoptosis and necrosis at least in parts of the kidneys (35). Since TNK1 can act as a molecular switch for determination of the TNF pathway and thus exacerbating apoptotic processes (24), a major pathological consequence of TNK1 overexpression seems to be disturbances in the renal epithelial cell barrier and tubular repair by proapoptotic features. Other reports postulate an anti-proliferative effect of TNK1 by inhibiting Ras/Raf signaling (36) and thus in the case of AKI, TNK1 activation could speculatively interfere with proliferation within healing processes.

However, the present study prompts further questions on the exact role of TNK1 in the kidney. Our cell culture data provide insight into the role of IL-6 being a potential driver for TNK1 expression; however, it remains unclear what the organ-wide effects of this upregulation may represent on a functional level. While we were able to demonstrate that blockade of complement decreases the expression of TNK1 and may prevent HS-induced AKI, blocking complement also significantly decreases the systemic inflammatory response, hindering a definition of the exact mechanistic processes involved. It is therefore subject of future studies to establish which role TNK1 plays in the pathophysiology of HS-induced AKI.

In conclusion, traumatic HS-induced systemic inflammation is reflected by enhanced IL-6 plasma levels among various species (mice, cynomolgus monkeys, and humans) and results in the development of early signs of AKI. TNK1 is detected in the blood shortly after trauma-HS and upregulated in the kidneys, which in non-human primates can be effectively blocked by complement C3 inhibition. *In vitro* data indicate that IL-6 rather than C3 cleavage results in upregulation of TNK1 in renal epithelial cells, suggesting that C3 inhibition *in vivo* indirectly neutralizes TNK1 as a potential driver of organ dysfunction via inhibition of the systemic inflammatory response. Further studies need to pinpoint the exact molecular mechanisms involved in these observations and address the therapeutic potential of direct TNK1 inhibition in the context of trauma and HS.

## Data Availability Statement

All datasets presented in this study are included in the article.

## Ethics Statement

The studies involving human participants were reviewed and approved by the Independent Local Ethics Committee of the University of Ulm, Germany. The patients/participants provided their written informed consent to participate in this study. The animal study was reviewed and approved by the Federal Authorities for Animal Research, Tübingen, Germany, and the IACUC of the Simian Conservation Breeding and Research Center in Makati City, Philippines.

## Author Contributions

RH, AP, PR, AK, TS, FG, JL, MG, and MH-L contributed to conception and design of the study. RH, EK, CB, SBr, AS, FS, SBü, AE, UW, RR, JT, MA, MK, MG, and MH-L performed the experiments and contributed to data collection. RH and MH-L performed the statistical analysis and wrote the first draft of the manuscript. PR, KN, BN, AK, TS, JL, and MG wrote sections of the manuscript. All authors contributed to manuscript revision, read, and approved the submitted version.

## Conflict of Interest

JL is the founder of Amyndas Pharmaceuticals, which is developing complement inhibitors (including third-generation compstatin analogs such as Cp40). JL is the inventor of patents or patent applications that describe the use of complement inhibitors for therapeutic purposes, some of which are developed by Amyndas Pharmaceuticals. JL is also the inventor of the compstatin technology licensed to Apellis Pharmaceuticals (i.e., 4(1 MeW)7 W/POT-4/APL-1 and PEGylated derivatives). The remaining authors declare that the research was conducted in the absence of any commercial or financial relationships that could be construed as a potential conflict of interest.
